# Tannic acid assisted metal–chelate interphase toward highly stable Zn metal anodes in rechargeable aqueous zinc-ion batteries

**DOI:** 10.3389/fchem.2022.981623

**Published:** 2022-08-10

**Authors:** Nan Hu, Hongyu Qin, Xiangyou Chen, Yanping Huang, Jing Xu, Huibing He

**Affiliations:** School of Chemistry and Chemical Engieering, Guangxi Key Laboratory of Petrochemical Resource Processing and Process Intensification Technology, Guangxi University, Nanning, China

**Keywords:** zinc-ion batteries, metal–chelate interphase, Zn anode, interfacial engineering, tannic acid

## Abstract

Aqueous zinc-ion batteries (AZIBs) have attracted extensive attention because of their eco-friendliness, intrinsic safety, and high theoretical capacity. Nevertheless, the long-standing Zn anode issues such as dendrite growth, hydrogen evolution, and passivation greatly restrict the further development of AZIBs. Herein, a metal–chelate interphase with high Zn affinity is constructed on the Zn metal surface (TA@Zn) via dipping metallic Zn into a tannic acid (TA) solution to address the aforementioned problems. Benefiting from the abundant hydrophilic and zincophilic phenolic hydroxyl groups of TA molecules, the metal–chelate interphase shows strong attraction for Zn^2+^ ions, guiding uniform zinc deposition as well as decreasing Zn^2+^ migration barrier. Therefore, the TA@Zn anode displays an extended lifespan of 850 h at 1 mA cm−^2^, 1 mAh cm^−2^ in the Zn|Zn symmetrical cell, and a high Coulombic efficiency of 96.8% in the Zn|Ti asymmetric cell. Furthermore, the Zn|V_2_O_5_ full cell using TA@Zn anode delivers an extremely high capacity retention of 95.9% after 750 cycles at 2 A g−^1^. This simple and effective strategy broadens the interfacial modification scope on Zn metal anodes for advanced rechargeable Zn metal batteries.

## 1 Introduction

Great efforts have been dedicated to developing the next-generation energy storage systems beyond lithium-ion batteries (Wan et al., 2022). Aqueous zinc-ion batteries (AZIBs) with metallic Zn anode are considered to be one of the most promising candidates because of the abundant resources, high theoretical capacity (820 mAh g^−1^/5,855 mAh g^−1^) of Zn, and intrinsic safety from the aqueous electrolytes (Liu et al., 2022c; Wang et al., 2022a). Nevertheless, the stubborn Zn anode issues such as dendrite growth, hydrogen evolution, and passivation not only limit the Coulombic efficiency (CE) of Zn stripping/plating but also result in a remarkably short lifespan and even an internal short-circuit, strictly hindering the movement of AZIBs from lab research to commercialization (Song et al., 2021).

To date, numerous strategies have been employed to address the aforementioned challenges, including surface engineering, electrolyte optimization, electrode design, and separator modification (Zhao et al., 2021; Li et al., 2022). Among them, surface engineering by building artificial interface layers has been regarded as a convenient but effective approach, as it can block the direct contact between the Zn anode and electrolyte, alleviating the water-induced side reactions, and also regulate the interfacial Zn^2+^ flux, suppressing the dendrite growth in the stripping/plating process (Tan et al., 2021; Wang et al., 2022b; Zhang et al., 2022a). However, most of the previously reported artificial interface layers lack stable adhesion onto the Zn metal, which are not compact enough to inhibit the side reactions during the whole cycling process (He et al., 2021; Zhao et al., 2021). Moreover, the weak adhesion of the interface layers is unable to tolerate the volume change during the long-term Zn plating process, which are easily peeled off from the Zn anode substrate, resulting the battery performance degradation (He et al., 2020; Wang et al., 2021). Therefore, it is crucial to develop an effective surface engineering layer for a stable and dendrite-free Zn anode (He and Liu 2020; Liu et al., 2022a).

Herein, by adopting tannic acid (TA) as the chelating linker, a metal–chelate interphase is constructed on the Zn anode surface (TA@Zn) to realize the stable electrode interface chemistry and guide the fast and homogenous Zn deposition. Different from the conventional artificial layers, this fabricated metal–chelate interphase is chemically anchored on the Zn substrate by the *in situ* complex reaction, showing high structure stability during the repeat Zn stripping/plating process. With the metal–chelate interphase, water dipoles are refused to be absorbed on the electrode surface, suppressing the water-induced side reactions (e.g., corrosion and hydrogen evolution reaction [HER]). In addition, the high zincophilicity endows the metal–chelate interphase with favorable Zn^2+^ absorption sites, leading to a balanced interfacial Zn^2+^ ion distribution and reduced desolvation energy, thus inducing fast and uniform Zn deposition. By virtue of these advantages, the symmetric cell with a modified Zn anode delivers a durable cycling life of 850 h under 1 mA cm^−2^, 1 mAh cm^−2^. The Zn|V_2_O_5_ full cell with TA@Zn anode also exhibits excellent cycling stability with an ultrahigh capacity retention of 95.9% over 750 cycles at 2 A g^−1^. This strategy might broaden the boundaries of the interfacial modification of metal anodes for advanced metal anode-based batteries.

## 2 Experimental

### 2.1 Materials

#### 2.1.1 Fabrication of metal–chelate interphase (TA@Zn)

A total of 5 g TA (Aladdin, 95%) was dissolved into 500 ml deionized water. The Zn plate (length × width: 10 × 5 cm) was then immersed in the transparent TA solution for 1 min. After being washed with deionized water and ethanol, the TA@Zn anode was obtained via vacuum drying at 60°C overnight.

#### 2.1.2 Synthesis of V_2_O_5_ cathode material

A total of 2 g commercial V_2_O_5_ (Aladdin, 99.0%) powder was added to 150 ml 2 M NaCl solution and stirred for 60 h under an ambient environment. Then, the precipitate was filtrated and washed with deionized water and ethanol several times. In conclusion, the precipitate was dried at vacuum under 60°C for 12 h, and the V_2_O_5_ cathode material was obtained.

#### 2.1.3 Characterizations

Attenuated total reflection flourier transformed infrared spectroscopy (ATR-FTIR) was conducted on the Nicolet 670 (United States). Field emission scanning electron microscopy was performed using SU8220 (Hitachi Corp, Japan). X-ray diffraction patterns were obtained on a D/Max-Ⅲ X-ray diffractometer (Rigaku Co., Japan) with Cu Kα radiation (*λ* = 0.15406 nm). Contact angles were measured on an SDC-350 contact angle meter (China). Cyclic voltammetry (CV), linear sweep voltammetry (LSV), and electrochemical impedance spectroscopy (EIS) tests were carried out on an electrochemical workstation (Interface 1010 E, Gamry, United States). Galvanostatic discharge–charge curves were tested by the NEWARE battery tester (MIHW-200-160CH, Shenzhen, China).

#### 2.1.4 Electrochemical measurements

CR2025-type coin cells were assembled for electrochemical test. A 2 M ZnSO_4_ aqueous solution was used as the electrolyte, whereas a piece of glass fiber was used as the separator in each cell. During the test, Zn metal was repeatedly plated/stripped between two identical Zn electrodes at a constant current (CC) density. For the rate test of the symmetric cell, the current densities were set at 0.5, 1, 2, 5, and 0.5 mA cm^−2^, respectively. The electrolyte dosage for each cell was 200 μL.

The Zn|V_2_O_5_ full cells were assembled with Zn foil as anodes, V_2_O_5_ electrode as the cathode, 2 M ZnSO_4_ as the electrolyte, and glass fiber as the separator. The CC mode was applied for the Zn|V_2_O_5_ cell tests, and the cut-off voltage was set as 0.4–1.6 V.

The EIS was conducted in a frequency range of 100 KHz to 0.1 Hz with an AC amplitude of 5 mV. The corrosion Tafel plots were tested between −0.7 and −1.3 V (vs. open-circuit voltage) at a scan rate of 1 mV s^−1^ in the 2 M ZnSO_4_ solution. The hydrogen evolution performance was performed between −0.9 and −1.6 V (vs. Ag/AgCl) at a scan rate of 1 mV/s. CV of the Zn||Zn symmetric cells was conducted in a voltage range of −0.1∼0.1 V; the CV of Zn|Ti asymmetric cells was conducted in a voltage range of −0.2∼0.2 V at a scan rate of 1 mV/s, in which Ti foil served as the counter and reference electrode; and the Zn|V_2_O_5_ full cells was conducted in a voltage range of 0.4 ∼ 1.6 V at a scan rate of 0.1 mV/s. Chronoamperometry (CA) curves were conducted at a constant overpotential of −150 mV for 500 s. All the electrochemical testing was conducted at a temperature of 25°C.

## 3 Results and discussion

**FIGURE 1:**
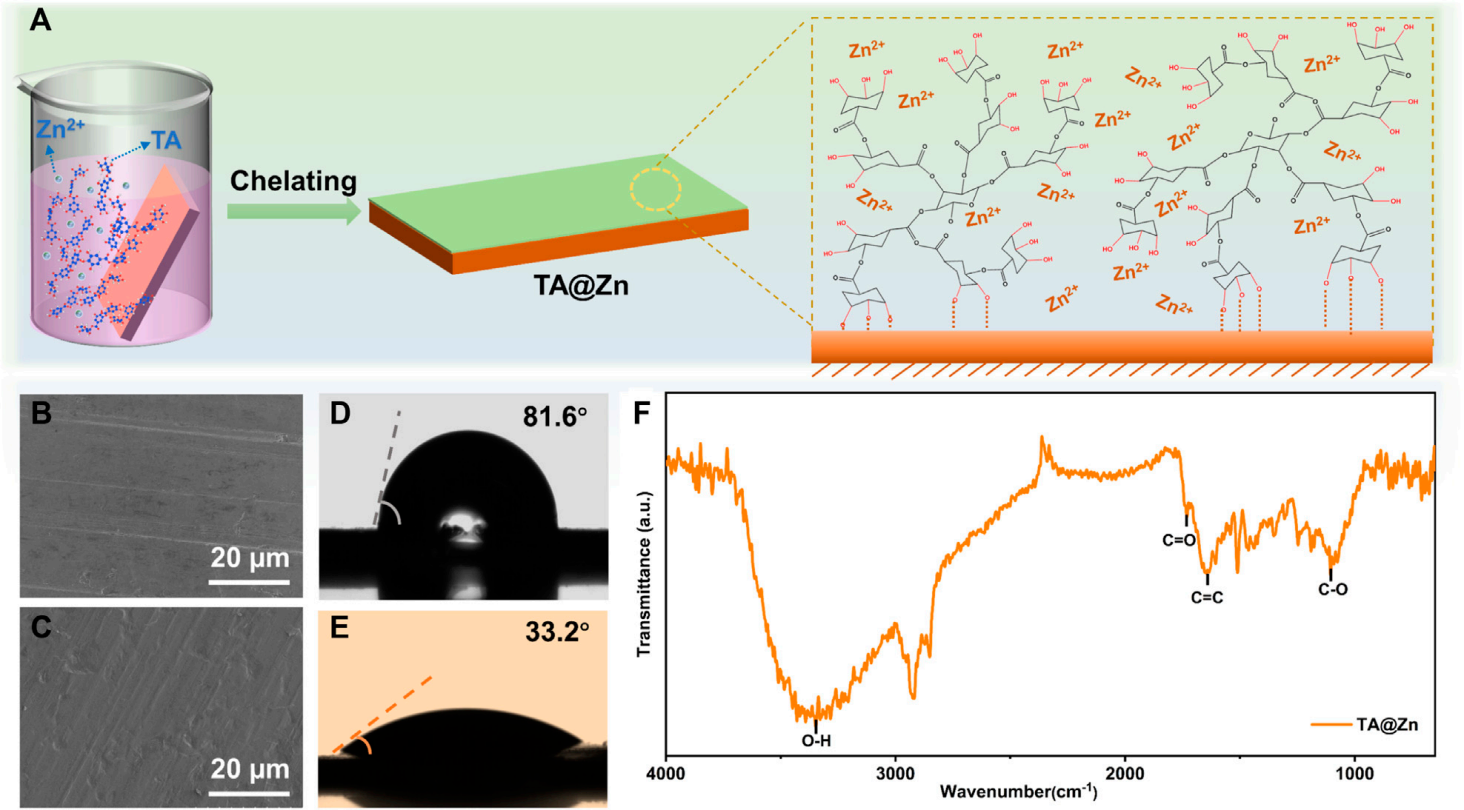
Fabrication and characterization of TA@Zn. **(A)** schematic illustration of the preparation process of TA@Zn. Scanning electron microscopy (SEM) images of **(B)** bare Zn and **(C)** TA@Zn after 50 cycles (100 h) at 1 mA cm^−2^ with an areal capacity of 1 mAh cm^−2^. Contact angle measurement of **(D)** bare Zn and **(E)** TA@Zn with 2 M ZnSO_4_ electrolyte. **(F)** ATR-FTIR spectrum of TA@Zn.

The formation process of the metal–chelate interphase is through a simple solution dip method (Figure 1A). As a natural phenolic compound, the abundant hydroxyl groups and aromatic ring of the TA molecule enable a strong adhesion between TA and various substances via covalent or noncovalent interactions, such as hydrogen bonding and π–π interaction (Yang et al., 2020). Moreover, the interaction between the free-pair electrons and the empty orbitals of Zn^2+^ ions could lead to the chelation reaction, thus forming a versatile metal–chelate interphase upon the Zn anode (TA@Zn). The existence of the metal–chelate interphase was examined using the scanning electron microscopy (SEM) images (Figures 1B,C), where the TA@Zn anode shows a thin and compact surface with no obvious morphology change compared with the bare Zn anode, showing that the formation of this metal–chelate interphase can barely alter the surface morphology, which will not affect the mass transportation at the electrolyte/electrode interface (Han et al., 2020). ATR-FTIR spectrum was then conducted to scrutinize the surface evolution of the Zn anode after introducing the metal–chelate interphase (Figure 1F). The board peak located at 3,354 cm^−1^ can be attributed to the characteristic hydroxyl group, and the peaks at 1727, 1,654, and 1,102 cm^−1^ can well be assigned to the C=O, C=C, and C–O stretching vibration from the TA molecule, respectively, indicating the successful fabrication of the metal–chelate interphase on Zn metal anode by our strategy (Zhou L et al., 2022).

**FIGURE 2:**
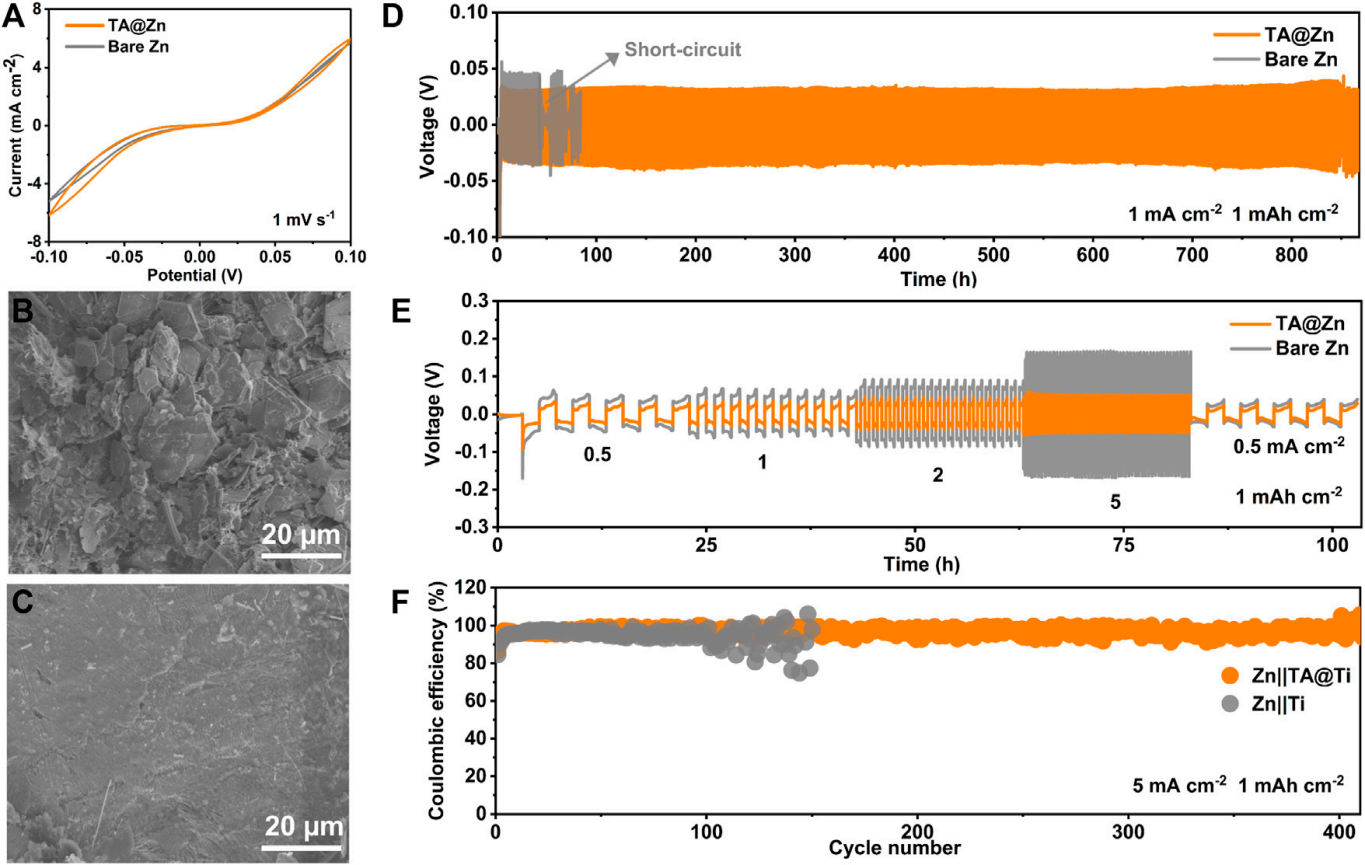
Electrochemical performance and characterizations of Zn symmetric cells. **(A)** cyclic voltammetry (CV) curves of the Zn symmetric cells at 1 mV s^−1^. SEM images of **(B)** bare Zn and **(C)** TA@Zn after cycled at 1 mA cm^−2^, 1 mAh cm^−2^. **(D)** long-term stability of Zn symmetric cells at 1 mA cm^−2^, 1 mAh cm^−2^. **(E)** rate performance of symmetric cells with a limited areal capacity of 1 mAh cm^−2^. **(F)** Coulombic efficiency of the Zn|Ti and Zn| TA@Ti cells at 5 mA cm^−2^, 1 mAh cm^−2^.

A contact angle was carried out to evaluate the hydrophilicity of the Zn anode surface (Figures 1D,E), as the wetting ability plays a key role in lowering the surface energy and promoting even Zn^2+^ flux, leading to fast and homogenous Zn deposition (Yuan et al., 2022). It is encouraging that the TA@Zn anode delivers a smaller contact angle of 33.3° compared with the bare Zn anode (88.6°), revealing an admirable surface wetting ability of the TA@Zn, which might bring a faster and uniform Zn deposition behavior (Zhou X. et al., 2022). The improved interfacial reactivity was also proved by the CV test. CV curves in Figure 2A show similar shapes for both bare Zn and TA@Zn anode, indicating the negligible effect of the metal–chelate interphase in the Zn^2+^/Zn redox process (Zhang et al., 2021). Moreover, the larger integrated peak area of the TA@Zn anode reflects the enlarged electrochemical active area as well as the enhanced Zn^2+^ concentration at the electrolyte/electrode interface, thus contributing to superior Zn^2+^ transfer kinetics in the Zn stripping/plating process (Hong et al., 2022).

The anticorrosion capability of the metal–chelate interphase was revealed using LSV (Supplementary Figure S1, Supplementary Table S1). The corrosion density of the TA@Zn (0.23 mA cm^−2^) is much lower than the bare Zn anode (4.45 mA cm^−2^), and the corrosion potential turns to a more positive position, suggesting the improved corrosion retardation of Zn anode via the metal–chelate interphase (Zhang Y et al., 2022). HER also supported the above LSV result (Supplementary Figure S2). The TA@Zn exhibits a larger HER overpotential (−1.11 V) than the bare Zn anode (−1.07 V), implying the better suppression capability toward HER enabled using the metal–chelate interphase (Ma et al., 2022).

To verify the positive effect of the metal–chelate interphase in the Zn stripping/plating process, the cyclic stability of the Zn|Zn symmetric cell was first tested at 1 mA cm^−2^, 1 mAh cm^−2^ (Figure 2B). It is certain that the symmetric cell with a bare Zn anode presents a larger voltage hysteresis (79.1 mV) (Supplementary Figure S3A) and suddenly failed after 45 h cycling. These phenomena may be attributed to the accumulation of side reaction products (Supplementary Figure S4) and the prominent dendrite growth (Figure 2B) (Zhou et al., 2021a; Zhou et al., 2022c). By sharp contrast, the TA@Zn|TA@Zn cell exhibits the best electrochemical performance with a prolonged cycling lifespan of over 850 h (Figure 2D) with a small voltage hysteresis of 57.8 mV at 1 mA cm^−2^, 1 mAh cm^−2^, implying that the immersion time of 1 min is the optimal condition for the fabrication of the TA@Zn electrode (Supplementary Figure S4). In addition, the TA@Zn anode exhibits a much smaller initial nucleation overpotential (0.076 V) than that of bare Zn (0.134 mV), suggesting favorable plating kinetics (Supplementary Figure S5). Furthermore, TA@Zn anode exhibits better cycling stability than bare Zn even at a high current density of 5 mA cm^−2^, 1 mAh cm^−2^ (Supplementary Figure S6). SEM images were then conducted to investigate the surface morphology evolution after the galvanostatic test. As shown in Figure 2B, the bare Zn anode surface was filled with disordered and irregularly shaped Zn dendrites, showing an extremely inhomogeneous Zn deposition, which might further deteriorate the interfacial stability, leading to the battery life degradation. In comparison, the TA@Zn anode exhibits dense and homogeneous Zn deposition even under the long-term cycling (Figure 2C; Supplementary Figure S7), implying the inhibited Zn dendrites growth by the constructed metal–chelate interphase (Qiu et al., 2022). Moreover, the TA@Zn electrode harvests better rate performance than the bare Zn at different current densities ranging from 0.5 to 5 mA cm^−2^ with a fixed areal capacity of 1 mAh cm^−2^ (Figure 2E). The symmetrical TA@Zn cell displays lower voltage hysteresis at all the currents, especially under high one of 5 mA cm^−2^. These results indicate that the formed metal–chelate interphase greatly improves the electrochemical performance of the Zn anode by lowing the deposition energy barrier and guiding the uniform Zn deposition. Meanwhile, the TA@Zn presents a high CE of 96.8% over 410 cycles (Figure 2F). By contrast, the CE of bare Zn|Ti holds random fluctuates after 100 cycles with a relatively low average value of 94.8%. In addition, the TA@Zn maintains continuously steady voltage hysteresis during 200 cycles, whereas bare Zn shows fluctuated voltage curves owing to the spontaneous side reaction and the formation of dendrites (Supplementary Figures S8, S9). The long cycle life coupled with the high CE of TA@Zn|Ti cells indicates reduced side reactions and improved reversibility with the assistance of the metal–chelate interphase (Liu et al., 2022c).

**FIGURE 3:**
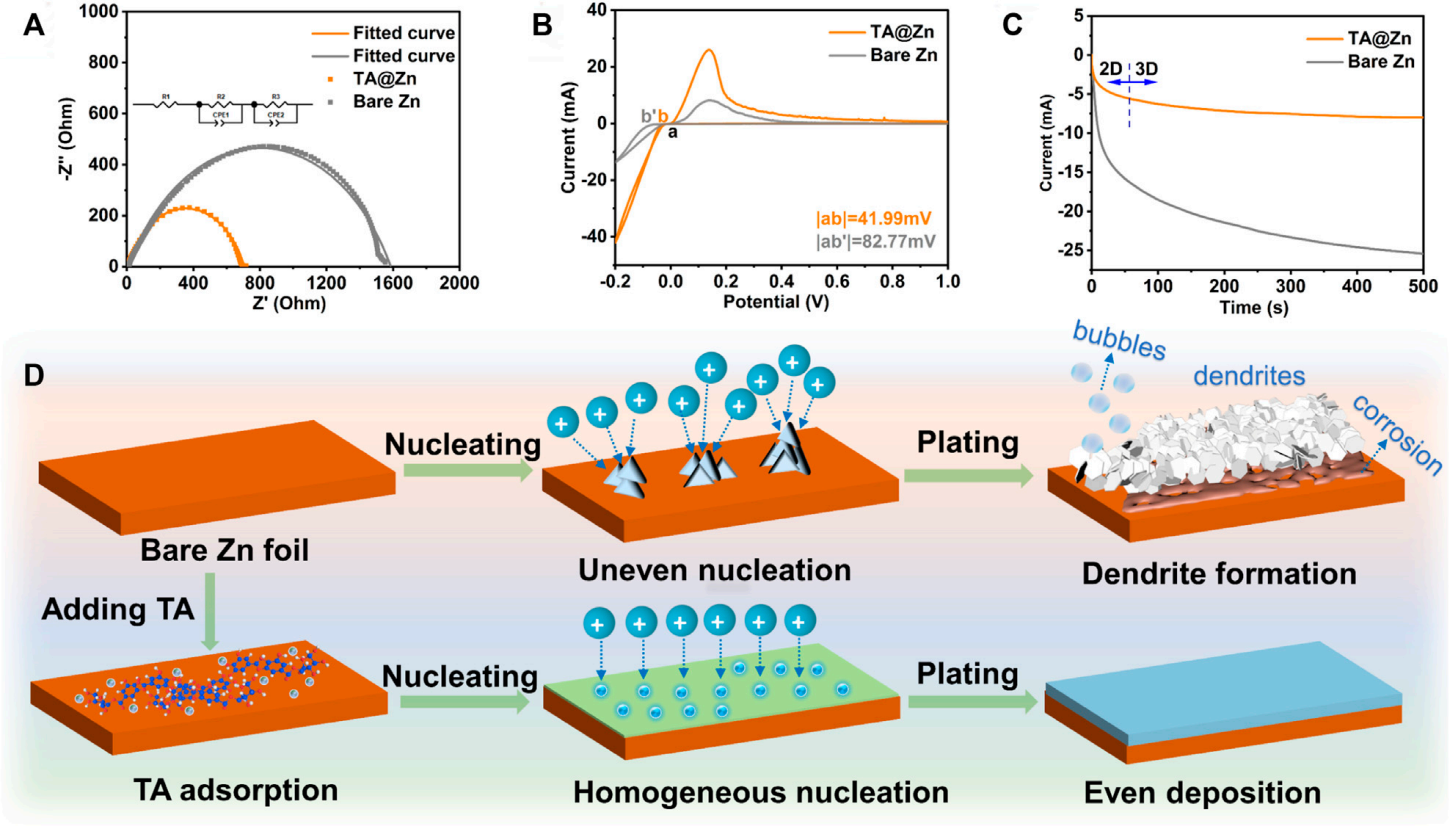
Zn plating behavior of bare Zn and TA@Zn. **(A)** electrochemical impedance spectra (EIS) of the Zn symmetric cells before cycled. **(B)** CV curves of the bare Zn|Ti and TA@Zn|Ti half cells. **(C)** chronoamperometric curves of symmetric cells using bare Zn and TA@Zn. **(D)** schematic illustration of Zn plating behavior on bare Zn and TA@Zn.

The underlying insights regarding the mechanism of metal–chelate interphase in regulating Zn deposition were carefully explored. EIS was employed to evaluate the charge-transfer resistance (*R*_*ct*_) of the electrode/electrolyte interface (Figure 3A). The TA@Zn electrode manifests a much lower *R*_*ct*_ of 482.9 Ω than bare Zn (1,129 Ω), revealing the favorable ion transfer kinetics at the electrode/electrolyte interface, which can facilitate the fast Zn deposition (Yang et al., 2022). Figure 3B shows the CV curves of the bare Zn|Ti and TA@Zn|Ti half cells. Compared with the bare Zn|Ti cell, the TA@Zn|Ti exhibits a much higher current density and lower nucleation overpotential of 41.99 mV, suggesting better electrochemical activity and fast kinetics at the metal–chelate interphase. The Zn nucleation and growth pattern were investigated via the CA test (Figure 3C). Under the applied overpotential of −150 mV, the bare Zn anode undergoes a continuous increase of current density up to 500 s, indicating the long and rampant 2D diffusion process, which can be ascribed to the aggregation of Zn nuclei and “tip-effect” driven growth of Zn dendrites. In comparison, the TA@Zn electrode exhibits a stable 3D diffusion after shorter 2D diffusion (within 50 s), reflecting the shielded 2D diffusion process on the metal–chelate interphase. During the 3D diffusion, the absorbed Zn^2+^ ions are locally reduced at the initial absorption sites, which is facile for the formation of dispersive nucleation sites, thus benefiting the uniform Zn deposition (Zhao et al., 2022).

Considering the above physical and electrochemical characterizations, the specific working mechanism of the metal–chelate interphase can be illustrated in Figure 3D. For the bare Zn electrode, the rough electrode surface usually generates uneven Zn^2+^ flux at the electrode/electrolyte interface, triggers the accumulation of the Zn nucleus, and acts as a charge-rich center for the further amplified growth of Zn dendrites (Zhou J et al., 2021). In addition, the enlarge exposed surface area would accelerate the HER rate and by-product formation, leading to a low CE and even battery failure (Liu et al., 2022b; Xiang et al., 2022). By contrast, with the metal–chelate interphase, Zn^2+^ ions are easily anchored on the cross-linked network of TA molecule, restricting the uncontrollable lateral diffusion of Zn^2+^ and thus producing homogenous nucleation sites for the even Zn deposition. Moreover, the strong coordination between metal–chelate interphase and Zn^2+^ ions enables fast desolvation kinetics of the hydrate Zn^2+^ ions (Zn(H_2_O)_6_^2+^), mitigating the water-induced issues. In a word, the introduced metal–chelate interphase kinetically improves the Zn deposition qualities to realize a stable and dendrite-free Zn anode (Liu et al., 2022a).

**FIGURE 4:**
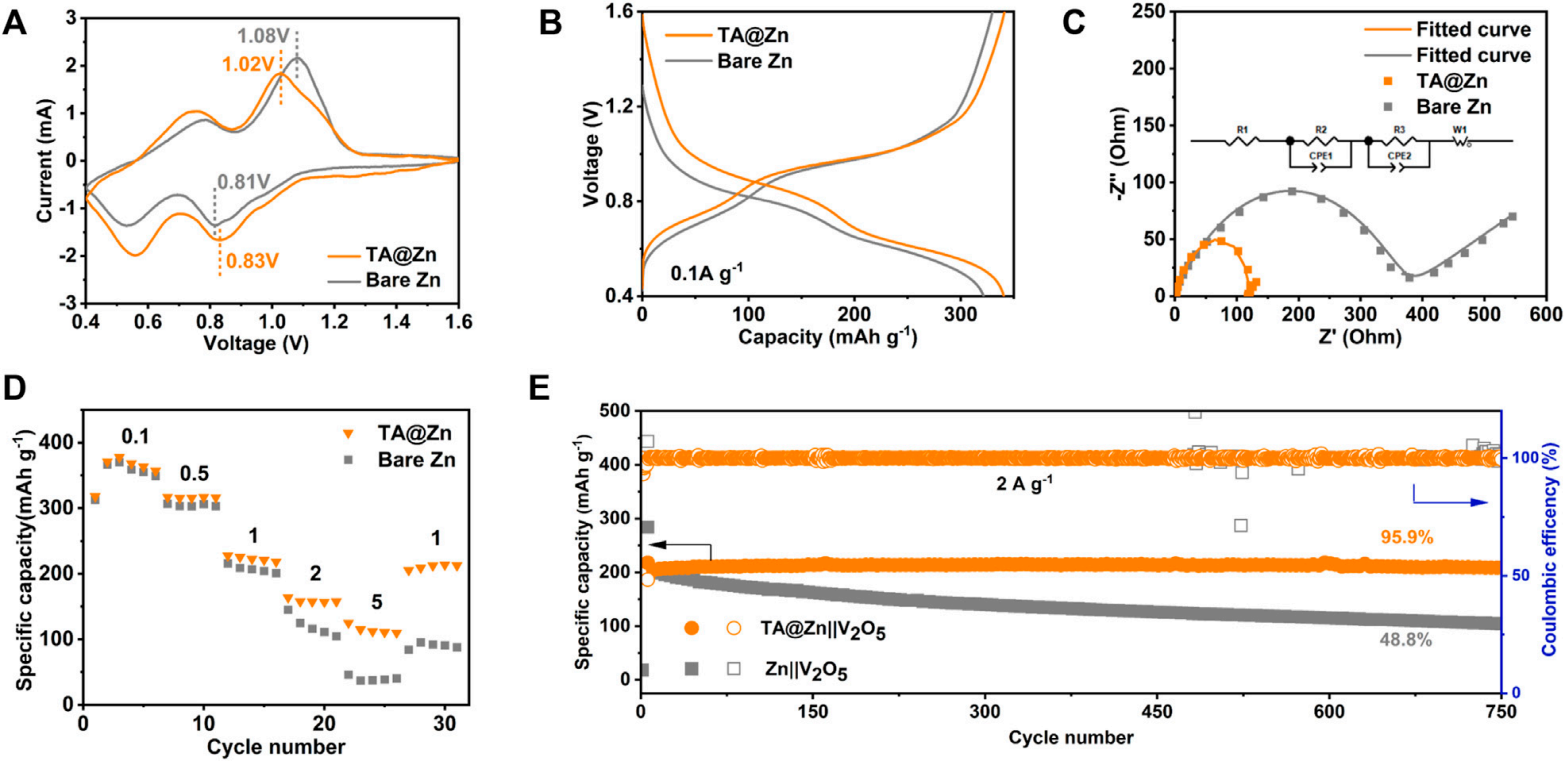
Electrochemical performance of full cells. **(A)** CV curves of Zn||V_2_O_5_ and TA@Zn||V_2_O_5_ full cells. **(B)** corresponding charge and discharge voltage profiles of bare Zn and TA@Zn at 0.1 A g^−1^. **(C)** EIS of Zn||V_2_O_5_ and TA@Zn||V_2_O_5_ full cells. **(D)** rate capabilities of the Zn||V_2_O_5_ at different current densities from 0.1 to 5 A g^−1^. **(E)** long-cycling performance of Zn||V_2_O_5_ of bare Zn and TA@Zn at 2 A g^−1^.

The feasibility of the TA@Zn anode in practical situations was certified by the Zn|V_2_O_5_ full cells with V_2_O_5_ as the cathodes (Supplementary Figure S10). Figure 4A illustrates the CV curves of the full cells using bare Zn or TA@Zn anode. The similar two redox pairs of two cells confirm the stability of the metal–chelate interphase during the electrochemical process without generating any new redox reactions, agreeing well with the stepwise plateaus in the typical discharge–charge profiles (Figure 4B) (Miao et al., 2022). Furthermore, a narrower voltage gap between the redox pairs of TA@Zn|V_2_O_5_ cell than bare Zn|V_2_O_5_ cell manifests better reversibility in the battery systems. The ion migration behavior in the full cells was revealed using the EIS (Figure 4C). The TA@Zn|V_2_O_5_ full cell delivers a much lower charge resistance of 110.1 Ω than the blank cell with bare Zn anode (331.8 Ω), which helps the rapid transportation. Because of the improved reaction kinetics, the TA@Zn|V_2_O_5_ full cell exhibits excellent rate performance (Figure 4D). In detail, when the current density reaches 5 A g^−1^, the TA@Zn|V_2_O_5_ cell still retains a high specific capacity of 114.4 mAh g^−1^ compared with the case of bare Zn|V_2_O_5_ cell (39.9 mAh g^−1^). In addition, the long-term cycling stability was also investigated (Figure 4E), in which the TA@Zn|V_2_O_5_ cell manifests an excellent cycling stability with a superhigh capacity retention of 95.9% after 750 cycles at 2 A g^−1^ (Figure 4E; Supplementary Figure S11), whereas the bare Zn|V_2_O_5_ cell demonstrates a low capacity retention of only 48.8%.

## 4 Conclusion

In summary, a metal–chelate interphase was built for targeting the notorious Zn anode issues to achieve stable and reversible Zn metal anodes. Because of the high affinity between Zn^2+^ and the TA molecule, the *in situ* anchored metal–chelate interphase provides sufficient coordination sites for Zn^2+^, enabling a fast and uniform Zn deposition. Moreover, the parasitic side reactions and dendrite growth can be suppressed using the metal–chelate interphase, thus leading to a dendrite-free Zn anode. As a result, the Zn anode with the metal–chelate interphase delivers a prolonged lifespan of 850 h at a current density of 1 mA cm^−2^ without dendrite formation. Furthermore, the Zn|V_2_O_5_ full cell exhibits an excellent electrochemical performance with a superior capacity retention of 95.9% after 750 cycles at 2 A g^−1^, showing great potential in practical application. Our proposed strategy of building metal–chelate interphase would develop advanced Zn-based rechargeable batteries and beyond.

## Data Availability

The original contributions presented in the study are included in the article/Supplementary Material; further inquiries can be directed to the corresponding author.
