# Supplementary material for: Tannic acid assisted metal–chelate interphase toward highly stable Zn metal anodes in rechargeable aqueous zinc-ion batteries
Source: Front Chem. 2022 Aug 10;10:981623. doi: 10.3389/fchem.2022.981623 (PMC9399369; doi:10.3389/fchem.2022.981623)
Supplement: Supplementary file 1 [file DataSheet1.docx]

Supporting Information

Tannic Acid Assisted Metal-Chelate Interphase towards Highly Stable Zn Metal Anodes in Rechargeable Aqueous Zinc-ion Batteries

Nan Hu^1^, Hongyu Qin^1^, Huibing He^1*^


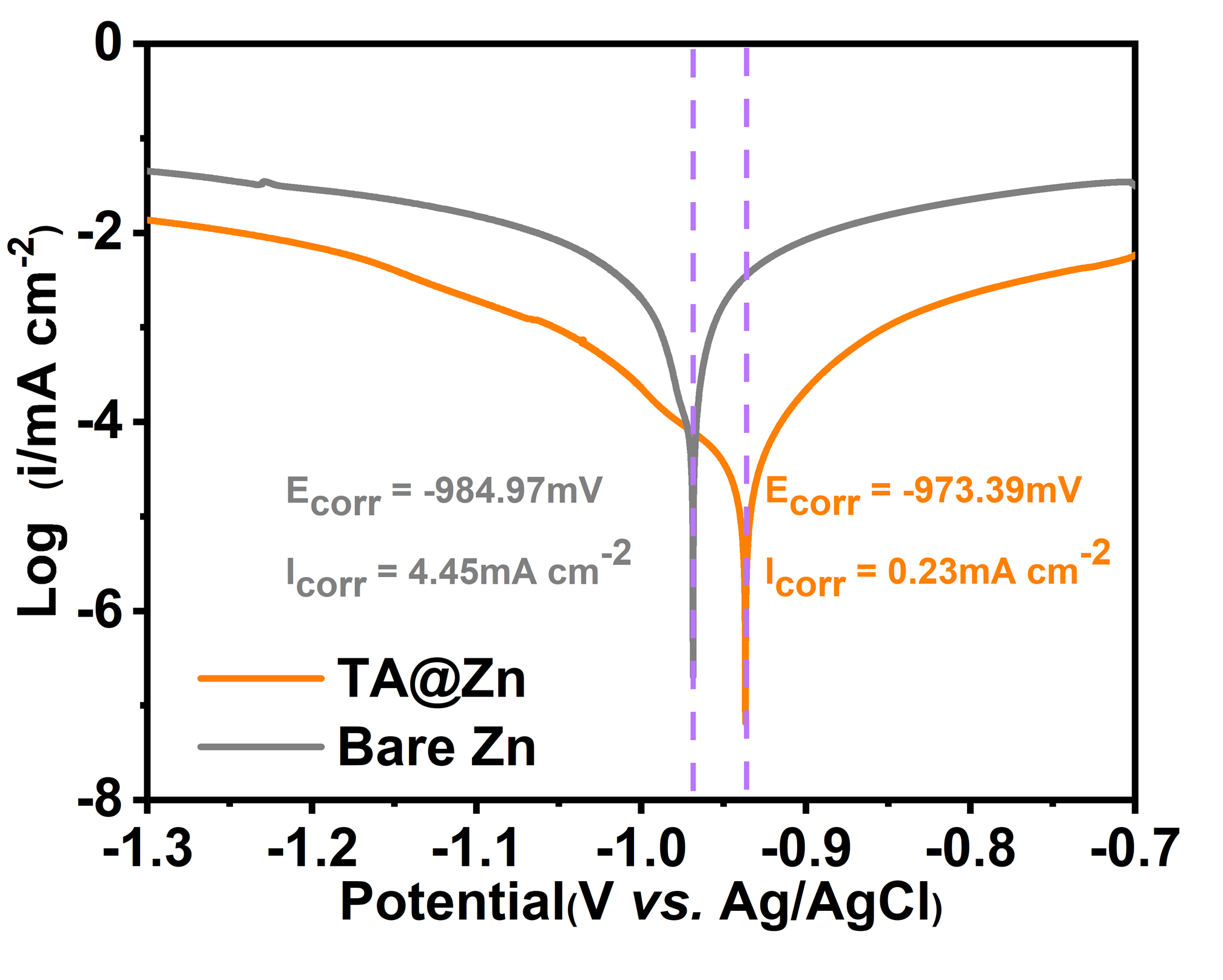


**Figure S1.** Comparison of Tafel plots representing corrosion behaviors of bare Zn electrode and TA@Zn electrode.


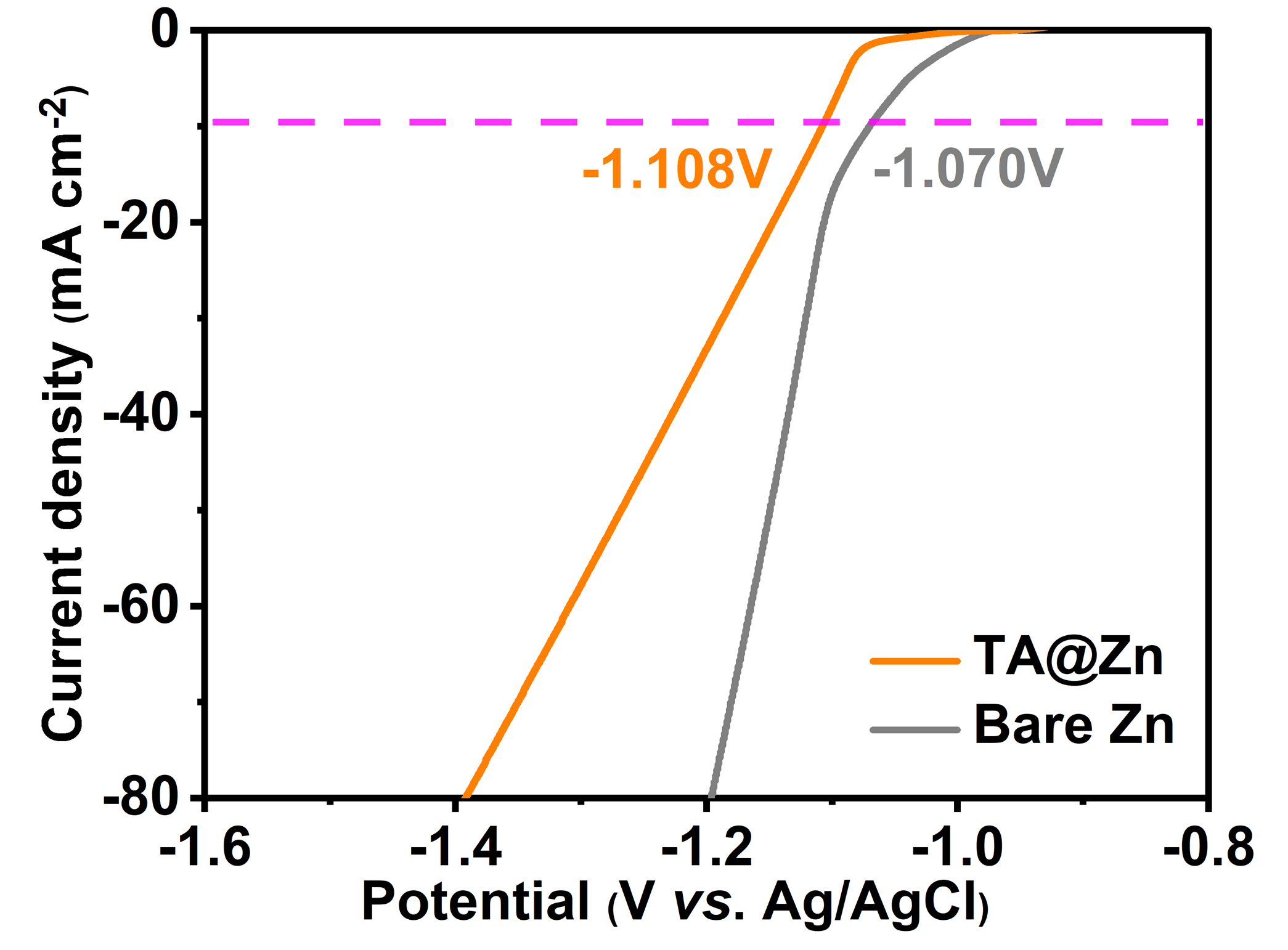


**Figure S2.** Hydrogen evolution reaction (HER) performance of bare Zn electrode and TA@Zn electrode.


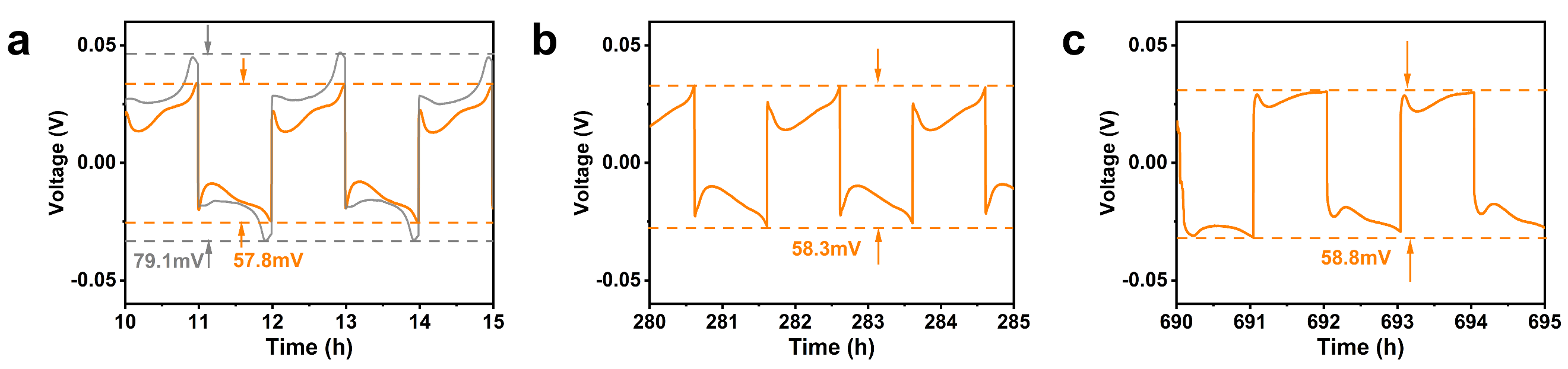


**Figure S3.** Selected voltage-time curves of Zn|Zn symmetric cell at different cycles at 1 mA cm^-2^, 1 mAh cm^-2^


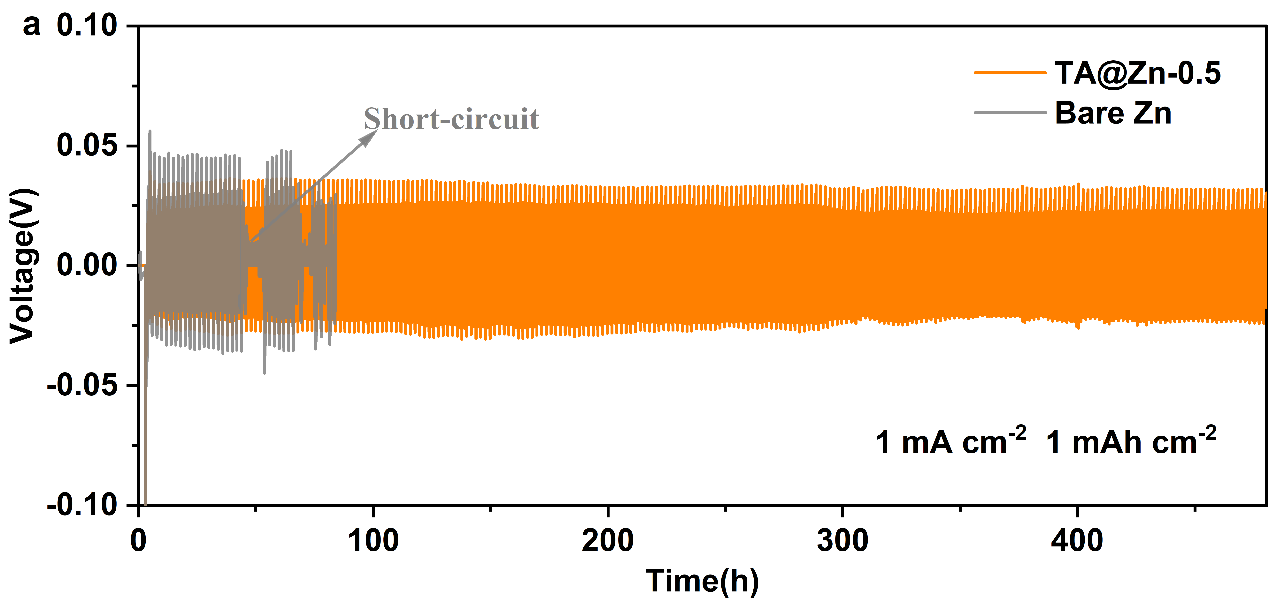


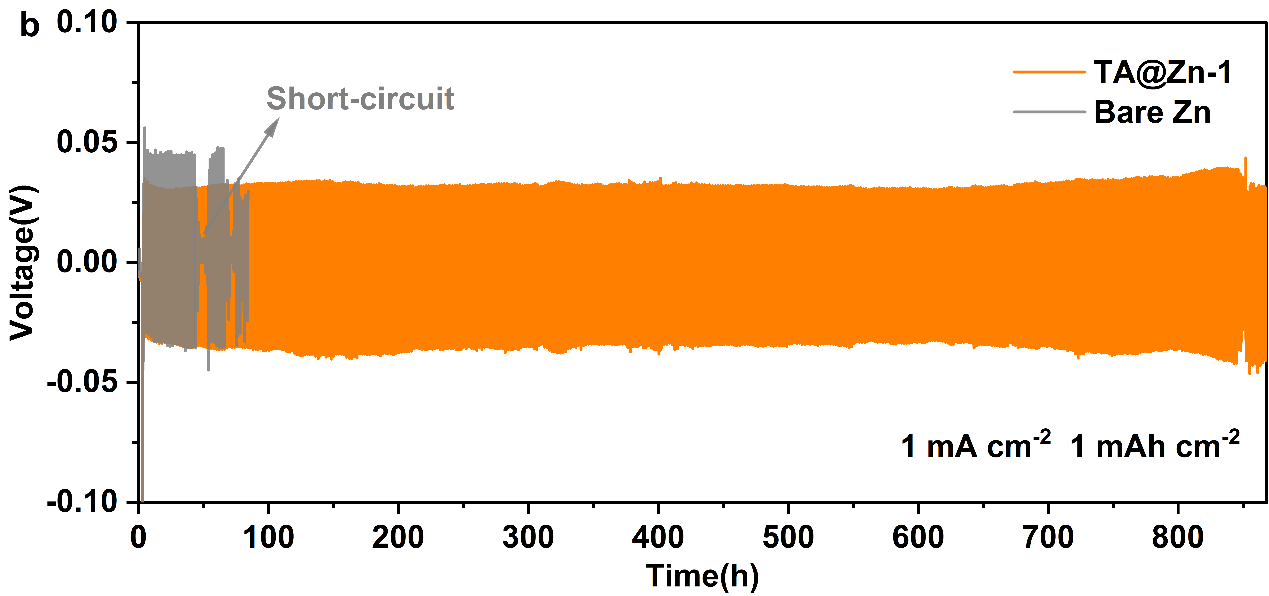


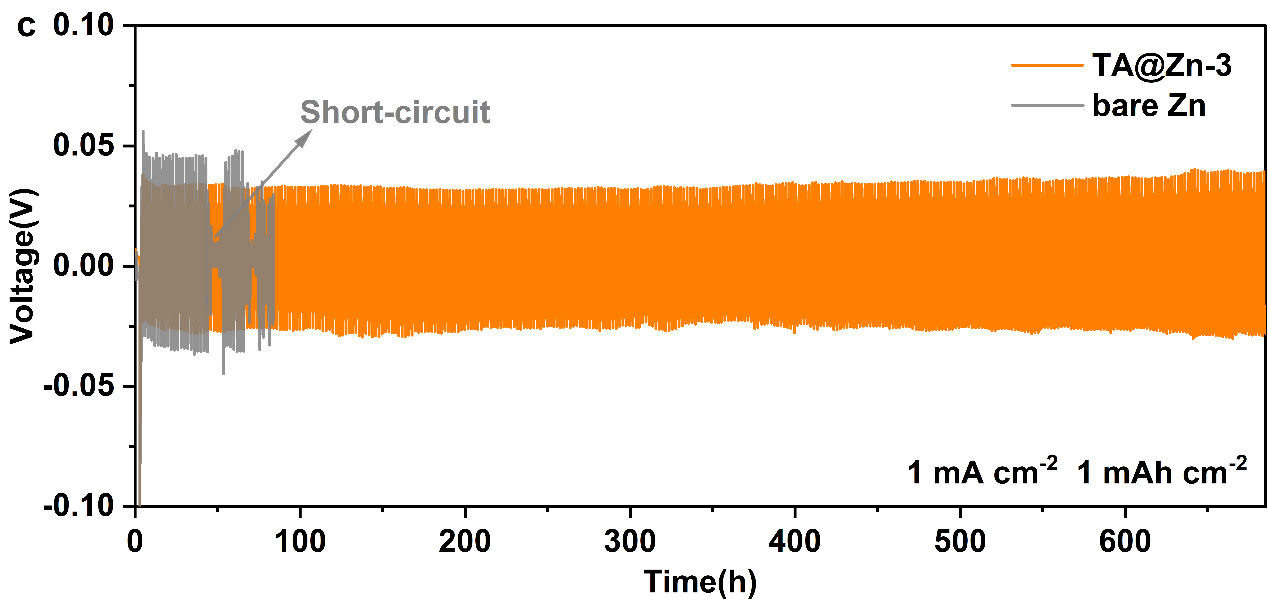


**Fig. S4** Long-term stability of Zn symmetric cells at 1 mA cm^-2^, 1mAh cm^-2^ by a soaking time of (a) 0.5 min, (b) 1min and (c) 3 min.


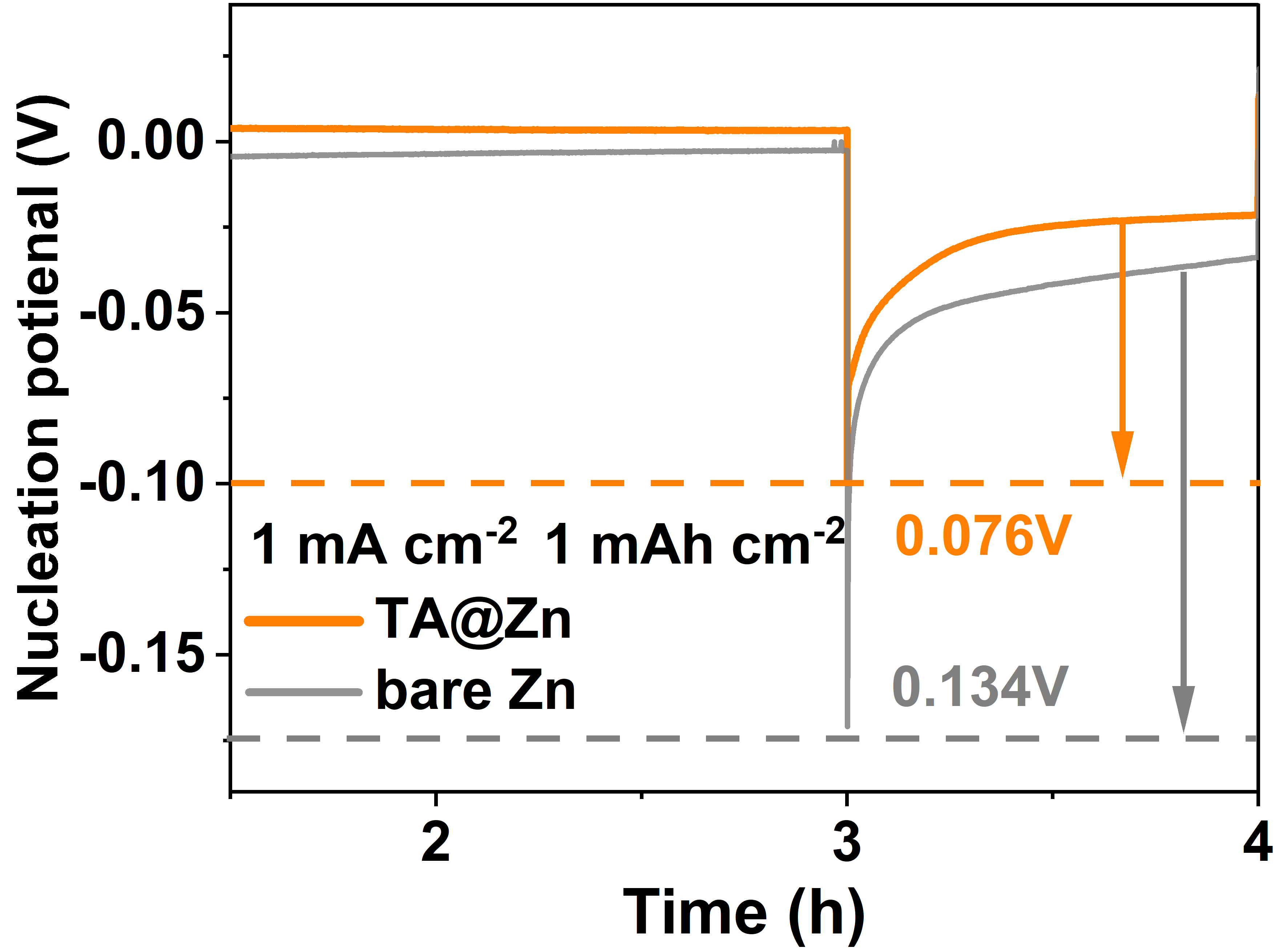


**Fig. S5** The initial nucleation overpotentials of TA@Zn and bare Zn anode at 1 mA cm^-2^, 1mAh cm^-2^.


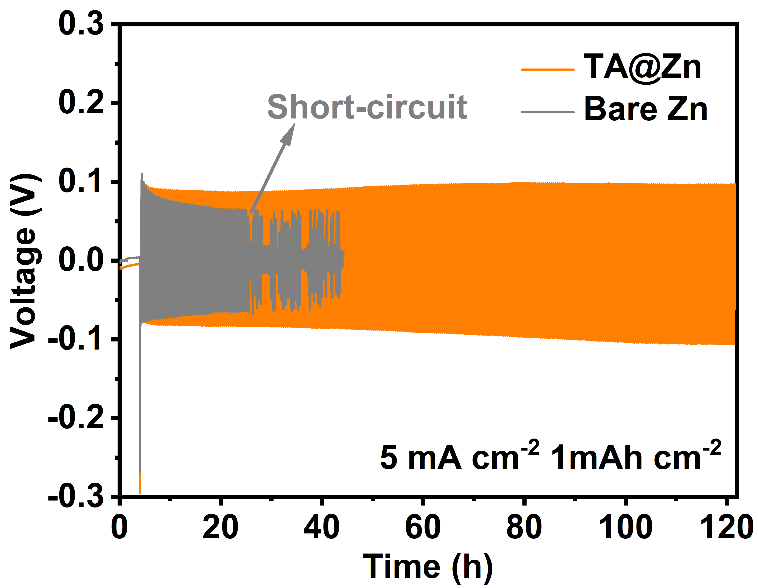


**Fig. S6** Zn stripping/plating ability for bare Zn and TA@Zn at high current densities of 5 mA cm^-2^ with a capacity of 1 mAh cm^-2^.


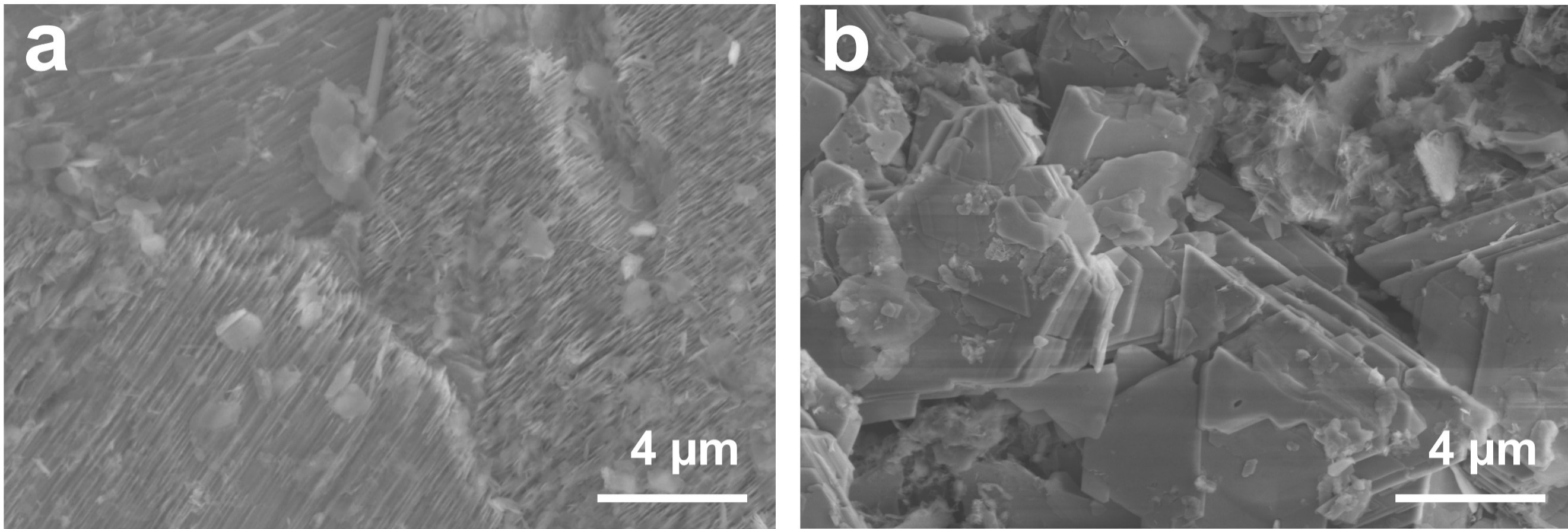


**Fig. S7** The SEM images of TA@Zn anodes (a) and bare Zn (b) after 50 cycles at 1 mA cm^-2^ with a capacity of 1 mAh cm^-2^.


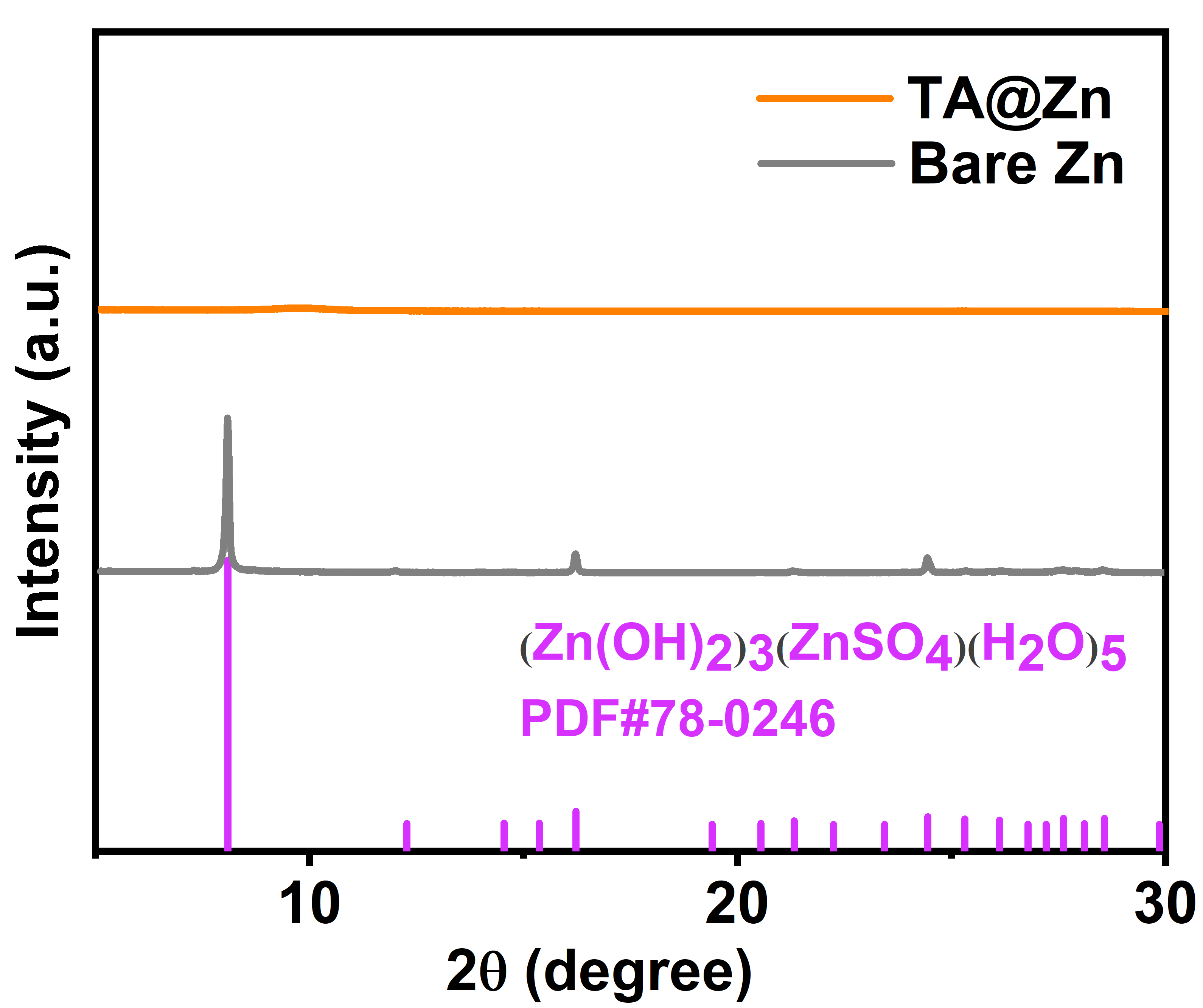


**Figure S8.** XRD characterization of bare Zn electrode and TA@Zn electrode in Zn symmetric cells after 20 cycles.


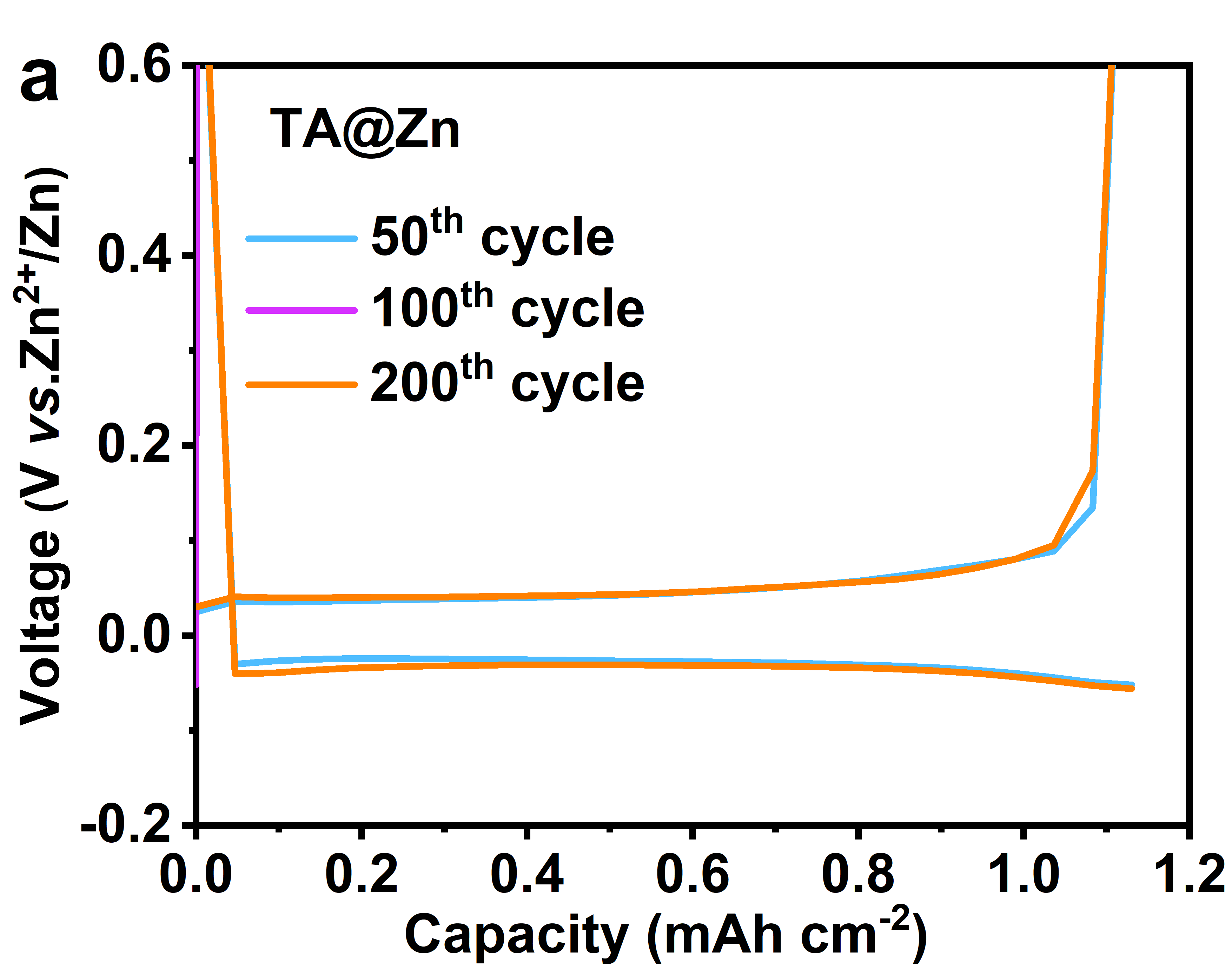

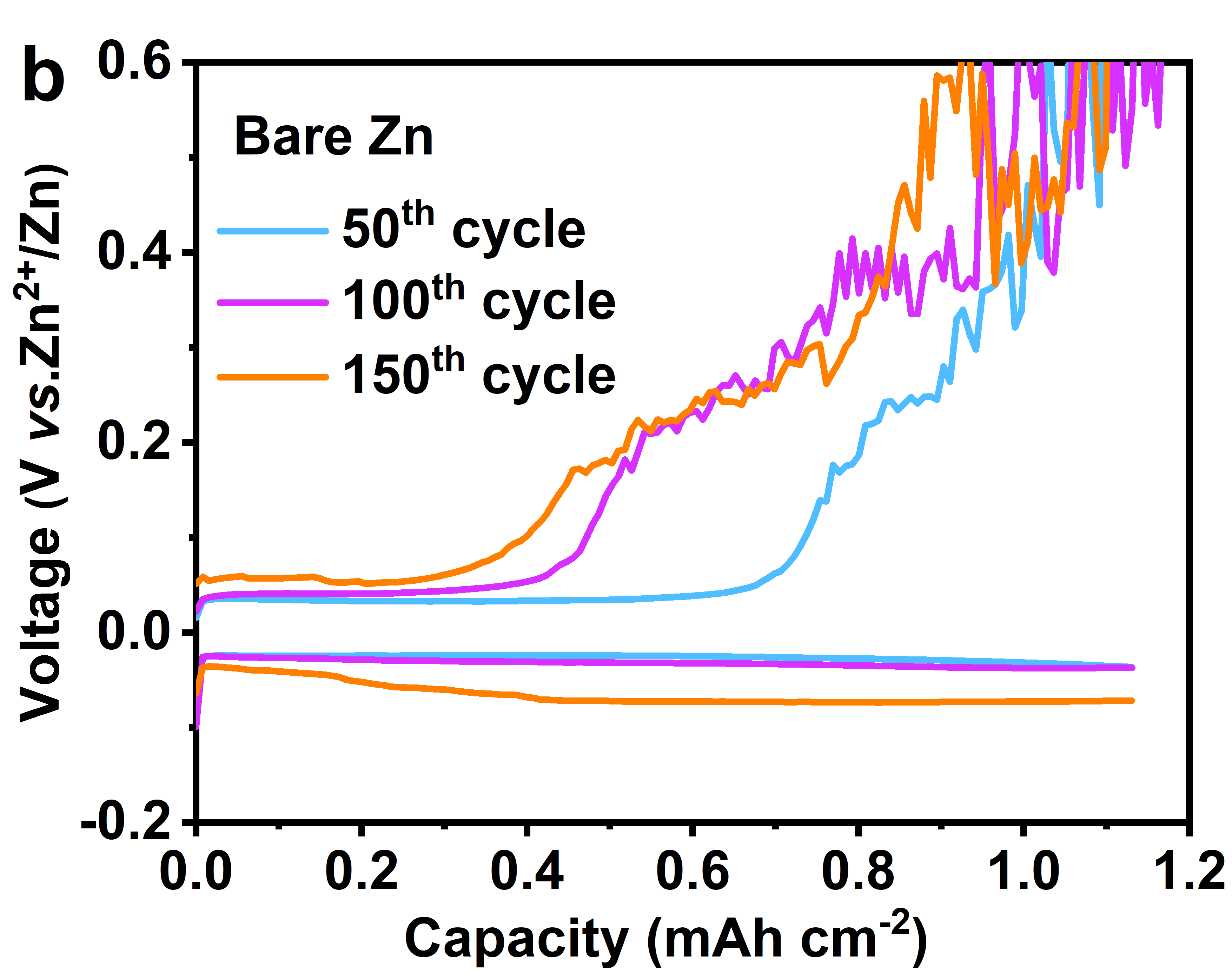


**Figure S9.** The voltage profiles of (a) the Zn||TA@Ti half-cell and (b) the Zn||Ti half-cell at 5 mA cm^−2^ 1mAh cm^−2^ at different cycles.


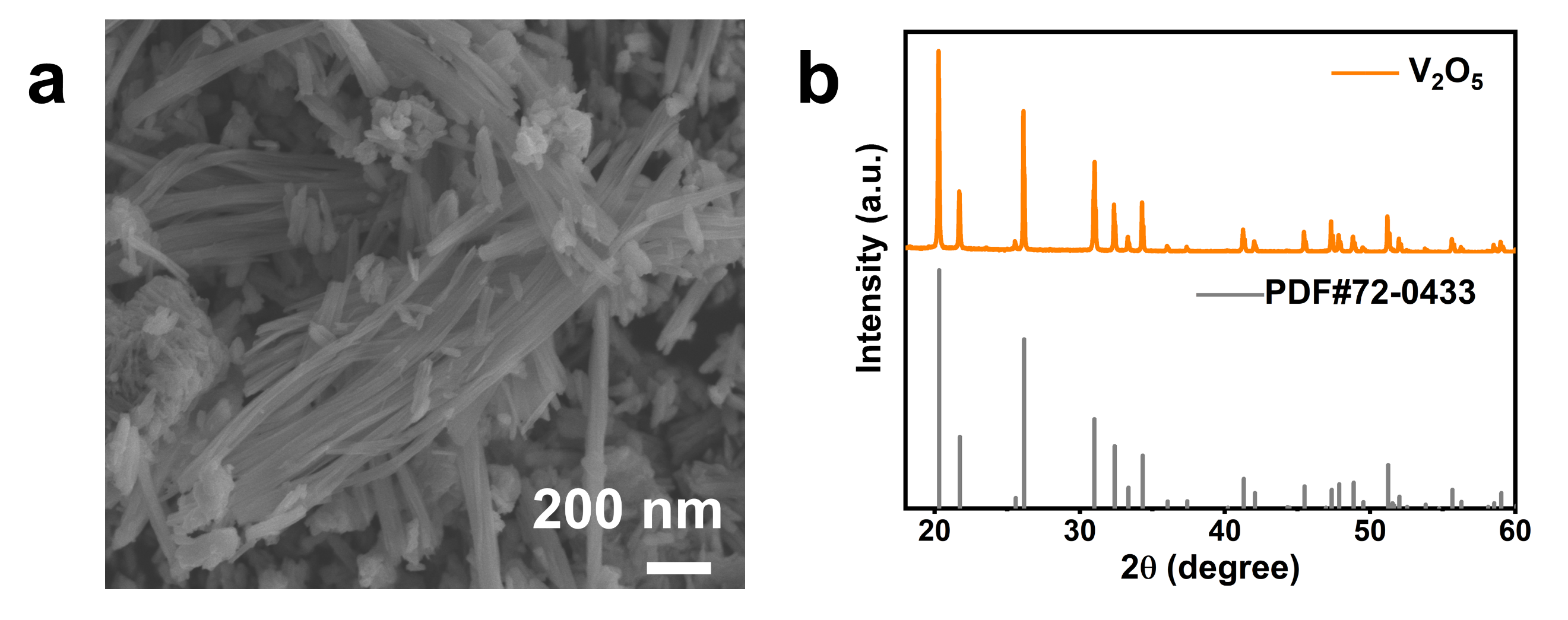


**Figure S10.** (a) SEM image (b)XRD pattern of the synthesized V_2_O_5_.


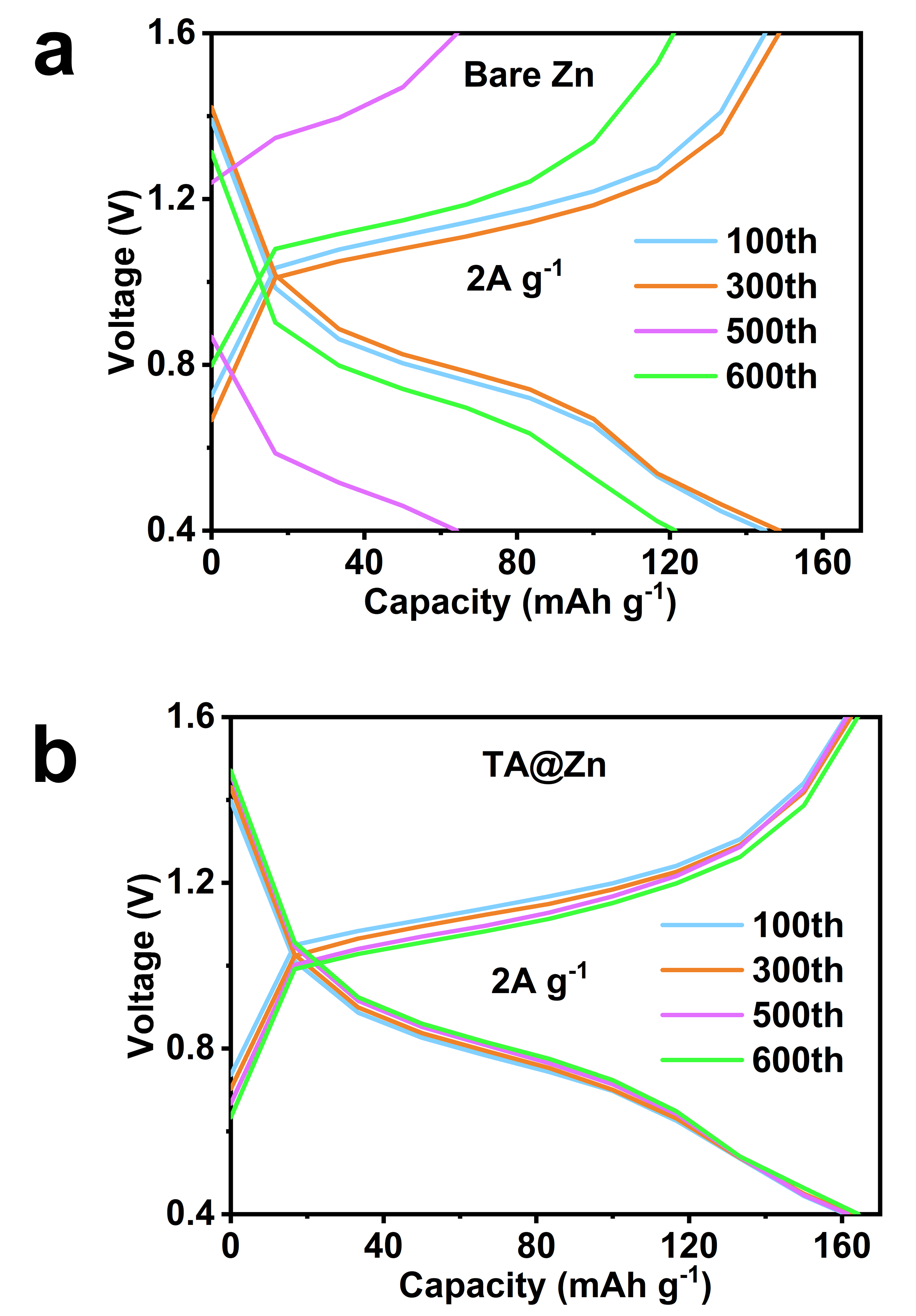


**Figure S11.** Charge-discharge voltage-capacity profiles of the Zn|V_2_O_5_ cells at 2A g^-1^.

**Table S1.** Fitting results of LSV.

| **Electrode** | **E_corr_ (mV)** | **I_corr_ (mA cm^-2^)** |
| --- | --- | --- |
| TA@Zn | -973.39 | 0.23 |
| Bare Zn | -984.97 | 4.45 |

**Table S2.** Fitting results of the EIS of Zn|Zn symmetric cells before cycling.

| **Electrode** | **R_1_(Ω)** | **R_2_(Ω)** | **R_3_(Ω)** |
| --- | --- | --- | --- |
| TA@Zn | 8.929 | 152.9 | 482.9 |
| Bare Zn | 9.718 | 443.3 | 1129 |

**Table S3.** Fitting results of the EIS of Zn|V_2_O_5_ and TA@Zn|V_2_O_5_ full cells before cycling.

| **Electrode** | **R_1_(Ω)** | **R_2_(Ω)** | **R_3_(Ω)** |
| --- | --- | --- | --- |
| TA@Zn | 2.479 | 8.9 | 331.8 |
| Bare Zn | 1.514 | 9.4 | 110.1 |
